# Forensic investigation of 23 autosomal STRs and application in Han and Mongolia ethnic groups

**DOI:** 10.1080/20961790.2018.1428782

**Published:** 2018-04-02

**Authors:** Xiang Sheng, Yali Wang, Jiashuo Zhang, Liqin Chen, Yuan Lin, Zhenmin Zhao, Chengtao Li, Suhua Zhang

**Affiliations:** a Shanghai Key Laboratory of Forensic Medicine, Shanghai Forensic Service Platform, Academy of Forensic Science, Shanghai, China; b Department of Forensic Medicine, Medical College of Soochow University, Suzhou, China; c Department of Forensic Medicine, Inner Mongolia Medical University, Hohhot, China

**Keywords:** Forensic genetics, short tandem repeats (STRs), Early Access Huaxia™ Platinum PCR kit

## Abstract

A forensic validation study of the Early Access Huaxia™ Platinum Polymerase Chain Reaction (PCR) kit was completed to document the performance capabilities and limitations. The genotyping of DNA samples was consistent across a large range of template DNA concentrations, with complete profiles obtained at 0.125 ng; however, no more than 2 mm × 1.2 mm punches of samples would be recommended for direct amplification. The size precision and accuracy test revealed the genotyping ability; while consistent results were obtained when comparing the kit with other commercially available systems. In addition, the whole PCR amplification can finish within approximately 45 min, making the system suitable for fast-detection. However, only partial profiles may be obtained with challenging samples, including DNA stored on Foam-Tipped Applicators (FTA) cards or some case samples. For the forensic application in ethnic groups, a total of 282 and 229 alleles were obtained in Han and Mongolia, respectively. Since the 23 short tandem repeats were independent from each other, the cumulative power of exclusion in duos was 0.999 999 157 188 and the cumulative power of exclusion in trios was 0.999 999 999 859 in the Han group while the cumulative power of exclusion in duos (CPE_duo_) was 0.999 998 848 26 and cumulative power of exclusion in trios (CPE_trio_) was 0.999 999 999 79 in the Mongolia group. And good internal consistency was found between the two investigated groups and the Sichuan Han, Hui, Tibetan and Uygur according to available reference data.

## Introduction

Short tandem repeats (STRs) have been the most common genetic markers used in forensic DNA analysis for the past 20 years because the multi-allelic nature of STRs produce many possible genotype combinations among individuals [1,2]. To facilitate the power of discrimination (PD), assist with the identification of a missing person, cut down adventitious matches and increase international data sharing, the Federal Bureau of Investigation declared that an additional seven loci (*D1S1656*, *D2S441*, *D2S1338*, *D10S1248*,* D12S391*, *D19S433* and *D22S1045*) would be added to the original Combined DNA Index System (CODIS), which included 13 Core Loci (*CSF1PO*, *D3S1358*, *D5S818*, *D7S820*, *D8S1179*, *D13S317*, *D16S539*, *D18S51*, *D21S11*, *FGA*, *TH01*, *TPOX*, *vWA*), at the beginning of 2017 [3]. The Early Access Huaxia™ Platinum Polymerase Chain Reaction (PCR) kit, which can simultaneously amplify the above mentioned 20 CODIS loci as well as *D6S1043* (specially selected for the Chinese population) [4], *Penta E* (allele number >10 in the Han ethnic group) [5] and *Penta D* (allele number >10 in the Han ethnic group) [5] were specially designed for the Chinese population by Thermo Fisher Scientific for the application of forensic parentage testing. For sex-determination, a Y-InDel (Insertion/Deletion) and Amelogenin were included. According to the marketing literature, this multiplex system has been optimized to work with purified DNA and protocols commonly used in forensic laboratories and is capable of a streamlined approach for database sample analysis by direct amplification of DNA collection cards with no need for purification.

To evaluate the actual forensic efficiency of the Early Access Huaxia™ Platinum PCR kit, forensic validation tests, including sensitivity, reliability and repeatability, sizing precision and accuracy, stutter analysis, case sample study and population investigation, were performed. The results obtained and reported here illustrate the performance capabilities and limitations of the multiplex system and how to obtain reliable results required for forensic casework and/or database analysis.

## Methods

All involved biological samples were collected upon approval of Ethics Committee at the Academy of Forensic Science. A written informed consent was obtained for each participant in this study. The main experiments were conducted at the Forensic Genetics Laboratory of the Academy of Forensic Science, which is an accredited laboratory by ISO 17025, in accordance with quality control measures. All the methods were carried out in accordance with the approved guidelines of the Academy of Forensic Science.

### Samples and experiment

The samples used in this study are listed in Supplementary Table S1.

Sensitivity test was performed with a serial dilution of control DNA 007 in triplicate. The template DNA amounts were 2, 1, 0.5, 0.25, 0.125 and 0.062 5 ng per reaction. A cycle number of 26 was adopted.

To determine whether the results are reliable and reproducible for comparison across laboratories, two separate standard laboratories (Shanghai Key Laboratory of Forensic Medicine, Academy of Forensic Science, Shanghai; and Forensic Biology Laboratory, Inner Mongolia Medical University, Inner Mongolia) tested the three control DNAs and 100 unrelated individuals in parallel. The same types of PCR thermal cyclers and genetic analysers were applied. All samples were previously genotyped with the Powerplex 21 System (Promega, USA) and the Goldeneye 20A Kit (Peoplespot, China). One hundred volunteers of Han ethnicity participated in the study with informed consent.

The sizing precision was evaluated by calculating the standard deviation in the size values. The size values were achieved from multiple injections (*n* = 24) of the Early Access Huaxia™ Platinum PCR kit allelic ladder. And the sizing accuracy study was performed by measuring the deviation in the size values obtained from alleles of 100 collected DNA samples from the corresponding allelic ladder. Both tests were performed in the Shanghai Key Laboratory of Forensic Medicine, Academy of Forensic Science, Shanghai. Additionally, the genotyping results of the 100 samples were also used for stutter analysis. The percentage of the stutter product was calculated by dividing the stutter peak height by the corresponding main peak height. The minimum threshold for the stutter peak height was set at 20 relative fluorescence units (RFU).

Samples including hair, buccal swab, finger swab and peripheral blood from three injured persons of a car accident were collected. Also, bloodstains on the leather seats, the airbag and the steering wheel were collected. The bloodstain was also collected from the white shirts and dark blue jeans of the three injured persons. To fully evaluate the direct amplification ability of the kit, the peripheral blood samples were prepared and dried on filter paper, Foam-Tipped Applicators (FTA) card, as well as cotton swab. DNA of hair, buccal swab, finger swab and peripheral blood was extracted and purified using the QIAamp DNA Mini Kit (Qiagen N.V., Netherlands) and quantified on a NanoDrop ND-1000 spectrophotometer. Then, 0.5 ng of each DNA sample was amplified. Other samples were directly amplified.

A mixed male/female DNA sample with known ratios (1:1, 1:3, 3:1, 1:9, 9:1, 1:19 and 19:1) for a total of 1 ng of template DNA was prepared for the mixture study.

Samples from 500 Eastern Chinese Han and 100 Mongolian individuals were collected for population studies. Informed consent was obtained prior to the study. Blood samples from these volunteers were collected on sterile filter papers. Samples were directly amplified in a full reaction volume.

And three positive controls (9947A, 9948 and 007) and negative controls (water and Chelex-100) were prepared for each run.

The PCR was prepared following the manufacturer's recommendations and performed using a GeneAmp PCR System 9700 thermal cycler (Thermo Fisher Scientific, USA) with “Max” mode. The final parameters for PCR were as follows: an initial denaturation step at 95 °C for 1 min, 26 cycles at 94 °C for 3 s, 59 °C for 16 s and 65 °C for 29 s. The whole PCR was finished within approximately 45 min. The PCR products were subsequently analysed by mixing 1 *µ*L of each amplified product, with 10 *µ*L of a 24:1 mixture of Hi-Di formamide (Thermo Fisher Scientific, USA) and GeneScan 600 LIZ Size Standard for electrophoresis on the 3500*xL* Genetic Analyser (Thermo Fisher Scientific, USA) using the specified J6 Matrix Standards. Samples were injected at 1.2 kV for 24 s and electrophoresed at 13.0 kV for 1 500 s with a run temperature of 60 °C. Data genotyping was performed using GeneMapper® ID-X Software v1.4 (Thermo Fisher Scientific, USA) with a recommended calling threshold of 175 RFU, indicating that the noise or stutter alleles below 175 RFU will be silent.

### Statistical analysis

The Hardy–Weinberg equilibrium (HWE), allelic frequencies, observed heterozygosity, expected heterozygosity, PD, power of matching and polymorphism information content (PIC) of the 23 autosomal STRs were calculated using Modified PowerStat.xls, and gene diversity (GD) was calculated as GD = *n*(1 − ∑*P_i_*^2^)/(*n* − 1), where *P_i_* is the frequency of the allele and *n* represents the total number of tested samples [6,7].

Peak height ratios (PHRs) of heterozygote were used to evaluate the intra-locus balance. Within a heterozygous locus, the lower peak height of the two alleles was divided by the higher peak height. Average peak heights were calculated by taking an average of the heterozygous peak heights in each marker and dividing the homozygous peak heights by two.

## Results and discussion

### Sensitivity testing

**Figure 1.**
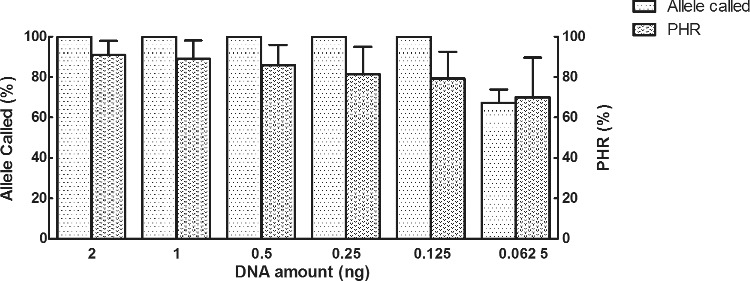
Information of called alleles and PHR of control DNA 007 ranging from 2 to 0.0625 ng.

For the amounts of the control DNA 007 ranging from 2 ng down to 0.062 5 ng, full profiles were observed from 2 to 0.125 ng. Allele dropouts were observed with 0.062 5 ng DNA. With 0.062 5 ng DNA, 67.39% of STRs were called (Figure 1). Mean PHR values of the observed heterozygotes ranged from 90.86% (2 ng) to 70.07% (0.062 5 ng) (Figure 1). As the quantity of DNA template decreased, the average peak heights gradually decreased from 8 977 to 245 RFUs; there was a codependent increase in variability and stochastic effects seen in the data as well. Therefore, the lower limit of DNA template for the kit in these experiments was 0.125 ng. Lower amounts of DNA may be detected with more PCR cycles than the 26 used in these experiments.

In addition, we also examined the sensitivity of the Early Access Huaxia™ Platinum PCR kit by utilizing direct amplification of the 15 blood samples collected on gauze. With direct amplification of blood samples, 100% of alleles were detected through one and two punches of 1.2 mm^2^ discs, and 72.15% of STRs were detected with three punches. As the number of punches increased, inhibitors existing in the samples began to overwhelm the amplification reaction reagent, thereby causing the allele dropouts [6,7].

### Reliability and repeatability study

For the reliability and repeatability study, control DNAs (007, 9947A and 9948) and blood from 100 Han individuals were tested in two involved laboratories. For the control DNAs, genotypes were accurate while compared to those provided in product manual and previous studies [6,7]; and consistent results were obtained in the laboratories. For the blood samples of the 100 Han individuals, a 1.2 mm^2^ disc of each was punched and added to the reaction well containing 25 *μ*L PCR mix without washing, extracting or quantifying. Full and concordant profiles of each individual were obtained from the two labs. However, significant differences were observed with the average peak height (*P* = 0.019 8) of the 100 tested samples between different labs, operators and instruments, with different test sites.

And for the control DNA, 1 and 0.5 ng were further used for the full PCR system (25 *μ*L) and half-reaction system (12.5 *μ*L), respectively. Consistent genotypes were obtained, and the PHR values of detected heterozygotes satisfied the forensic application needs (>0.6). The above results indicated that the half-reaction volume with half the recommended DNA amount (0.5 ng) can be an effective and economic solution for DNA-database establishment.

Additionally, consistent results of the 100 samples were obtained when comparing the Early Access Huaxia™ Platinum PCR kit with Powerplex 21 System (Promega, USA) and the Goldeneye 20A Kit (Peoplespot, China).

### Sizing precision and accuracy analysis

Sizing precision study was explored by injecting allelic ladder on 24 capillaries on 3500*xL* Genetic Analyser (Thermo Fisher Scientific, USA). The average size of bases and standard deviation were calculated for each allele. The fragment sizes were plotted against the three standard deviations (Supplementary Figure S1). Results showed that the 3 × standard deviation (SD) of the allele size for multiple injections did not exceed 0.20. The lowest SD was 0.07415 at *D3S1358*, while the highest was 0.128 at *Penta D*.

The sizing accuracy assay showed that all observed alleles (*N* = 4 053) from the 100 individuals were within ±0.50 bases of the corresponding alleles in the allelic ladder (Supplementary Figure S2). The largest size difference was 0.41 bases, which was observed at the *Penta E* locus. The Supplementary Figures S1 and S2 show that this system is reliable and accurate for determining off-ladder alleles and microvariants.

### Stutter information

Stutter events are the result of slippage when DNA samples are amplified by PCR [6–9]. The presence of stutter peaks will complicate the interpretation of forensic samples. In this study, we calculated the minus and plus stutter (*N* − 3/*N* + 3 for trinucleotide STR marker *D22S1045*, *N* − 5/*N* + 5 for pentanucleotide markers *Penta D* and *Penta E* and *N* − 4/*N* + 4 for the rest of the tetranucleotide STR markers) for the included 23 autosomal STR loci. The stutter of count, maximum, minimum, mean, SD and the average stutters plus three SD values are shown in Table 1. The mean stutter ratios plus 3 × SD values are used as the stutter filters. The highest stutter filter of minus stutter was observed at *D10S1248* (15.54%), while the lowest was observed at *Penta D* (4.09%); the highest stutter filter of plus stutter was observed at *D1S1656* (19.46%), while the lowest was observed at *D10S1248* (4.20%).

**Table 1.** Stutter ratios for the Early Access Huaxia^TM^ Platinum PCR kit from 100 individuals.

	Minus stutter (%)						Plus stutter (%)					
STR	Count	Max	Min	Mean	SD	Mean + 3SD	Count	Max	Min	Mean	SD	Mean + 3SD
*D3S1358*	108	11.96	5.02	7.94	1.40	12.14	56	16.97	0.56	3.23	4.06	15.42
*vWA*	137	11.98	2.62	7.09	2.30	13.99	66	18.67	0.53	3.62	3.86	15.19
*D16S539*	130	10.52	3.46	6.23	1.65	11.16	92	12.17	0.51	2.71	2.84	11.22
*CSF1PO*	92	12.29	3.57	6.59	1.59	11.35	80	13.59	0.64	2.78	3.07	11.98
*TPOX*	112	4.94	1.63	2.91	1.01	5.94	11	3.66	0.38	2.02	1.47	6.42
*D8S1179*	140	11.38	0.42	7.64	1.54	11.64	65	12.33	0.81	4.27	3.52	14.84
*D21S11*	122	14.70	1.01	8.64	2.01	14.66	90	13.07	0.43	2.52	2.94	11.34
*D18S51*	146	18.27	4.73	7.72	2.44	15.03	84	13.47	0.54	2.76	3.15	12.22
*Penta E*	147	15.99	0.83	4.21	1.82	9.66	43	8.53	0.46	2.53	2.14	8.95
*D2S441*	130	8.22	0.79	5.55	1.44	9.86	77	18.13	0.54	3.09	3.27	12.89
*D19S433*	134	12.23	3.05	7.05	1.53	11.64	28	10.34	0.54	4.03	3.90	15.73
*TH01*	121	5.56	0.47	2.85	0.74	5.08	39	7.89	0.22	2.95	1.50	7.46
*FGA*	138	12.90	4.03	7.92	1.67	12.93	66	11.65	0.58	3.09	3.57	13.81
*D22S1045*	128	21.50	0.76	7.90	3.99	9.88	107	20.95	0.84	4.80	3.45	15.14
*D5S818*	122	9.80	2.48	6.19	1.50	10.67	91	11.80	0.80	3.20	3.00	12.20
*D13S317*	130	9.78	1.97	4.78	2.01	10.81	75	8.17	0.41	1.83	1.87	7.45
*D7S820*	123	10.17	2.42	4.98	1.57	9.70	58	8.77	0.49	2.70	2.83	11.20
*D6S1043*	165	10.66	0.65	7.19	1.58	11.93	118	10.93	0.73	2.35	2.60	10.14
*D10S1248*	130	19.32	9.29	8.46	2.36	15.54	60	19.08	0.49	5.30	4.72	19.46
*D1S1656*	117	14.47	1.88	8.59	2.02	14.64	90	13.68	0.74	3.08	3.47	13.49
*D12S391*	120	13.55	3.99	8.82	1.86	14.41	57	16.11	0.44	4.20	4.49	17.68
*D2S1338*	137	17.33	3.37	8.80	2.06	14.99	35	16.49	0.34	4.32	4.56	18.00
*Penta D*	110	4.99	0.64	1.82	0.76	4.09	22	3.30	0.40	1.70	0.83	4.20

### Testing of case-type samples

Table 2 lists the genotyping detail of involved case samples in this study. Biological samples collected from the three injured persons including hair, buccal swab, finger swab and peripheral blood were fully genotyped; the genotypes of different biological samples of each individual were consistent; and the average peak height and PHR values of heterozygotes can satisfy the forensic application demands. Each profile of the three individuals was used as reference. For samples collected at the accident scene, partial DNA profiles were obtained with bloodstain on the dark blue jeans, airbag and leather seats. And especially with the obtained heterozygotes from the bloodstain on the dark blue jeans, the averaged PHR value was 0.57, which is lower than the recommended limit of allelic balance of heterozygotes [6]. And when we prepared peripheral blood on three different collecting materials, lower genotyping efficiency of FTA cards was observed with the direct amplification method. Larger loci were typically the first to drop-out. The above results indicate that PCR inhibitors present in forensic evidentiary samples may reduce the efficiency in amplifying some loci and/or alleles, resulting in an imbalance in the signal obtained across the DNA profile. Further optimization such as improving magnesium concentrations in their PCR buffer may help overcome PCR inhibitors.

**Table 2.** Detected information of case samples from three injured persons of a car accident.

Type	Samples	Calling rate (%)	Average peak height (RUF)	Average PHR	Note
Biological samples collected from individuals	Hair	100	2 584	0.81	0.5 ng of extracted DNA for PCR
	Buccal swab	100	3 126	0.79	0.5 ng of extracted DNA for PCR
	Finger swab	100	1 983	0.78	0.5 ng of extracted DNA for PCR
	Peripheral blood	100	2 309	0.83	0.5 ng of extracted DNA for PCR
Blood samples collected from the accident scene	Bloodstain on white shirt	100	2 403	0.77	Direct amplification; 1.2 mm^2^
	Bloodstain on dark blue jeans	75.36	1 398	0.57	Direct amplification; 1.2 mm^2^
	Blood on airbag	84.06	1 602	0.76	Direct amplification; 1.2 mm^2^
	Blood on leather seats	95.65	2 011	0.73	Collected on cotton swab; direct amplification; 1.2 mm^2^
	Blood on steering wheel	100	1 753	0.88	Collected on cotton swab; direct amplification; 1.2 mm^2^
Blood samples collected on different materials	Blood on filter paper	100	2 531	0.91	Manual prepared with peripheral blood; direct amplification; 1.2 mm^2^
	Blood on FTA card	86.96	1 652	0.62	Manual prepared with peripheral blood; direct amplification; 1.2 mm^2^
	Blood on cotton swab	100	2 397	0.87	Manual prepared with peripheral blood; direct amplification; 1.2 mm^2^

For sex determination, small PCR fragments of Amelogenin and the Y-InDel of all involved case samples were fully detected.

### Mixture study

**Figure 2.**
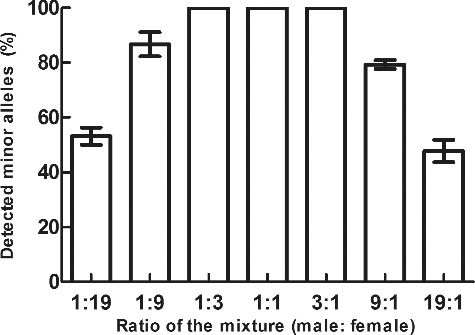
The mixed male/female DNA samples with known ratios amplified with the Early Access Huaxia™ Platinum PCR kit using the recommended protocol. Averaged percentages of detected minor alleles were against ratios of the mixture. Error bars represent the plus and minus standard deviations in triplicate.

Mixtures are common forensic samples and mixture study is useful to mixture interpretation, including the number of contributors to the mixture, the major and minor contributor genotypes and contributor ratios or proportions [6]. Here, the mixed male/female DNA samples with known ratios were tested in triplicate. The detected percentages of minor alleles were calculated for each mixture (Figure 2). All of the minor alleles were called for ratios of 1:1, 1:3 and 3:1. Some minor alleles dropped out in the 1:9 and 9:1 mixtures, resulting in (86.65 ± 4.47)% and (79.28 ± 1.56)% of the minor alleles called, respectively. And when the mixture ratio was increased to 1:19 and 19:1, an average of (53.13 ± 3.13)%, (47.75 ± 4.13)%, respectively, of the minor alleles were called. The minor component of 1:19 and 19:1 mixtures is 0.005 ng which is lower than the detection limit (0.125 ng) discovered through the sensitivity testing. As expected, the results indicate that as the mixture ratios became higher, there was a decrease in the percentage of minor alleles that could be identified.

### Forensic investigation in Han and Mongolia ethnic groups

Population studies were performed on Eastern Chinese Han and Mongolia ethnic groups. All 23 autosomal STRs follow the HWE in these populations after Bonferroni correction (Supplementary Table S2). The forensic parameters of the two ethnic groups are listed in Supplementary Table S2.

In the Eastern Chinese Han group, a total of 282 alleles were found, and the highest number of alleles (*n* = 29) was observed at *Penta E*. The highest PD value was 0.986 2 at *Penta E*, while the lowest was 0.791 5 at the *TPOX* locus. The highest PIC value was 0.914 0 at *Penta E*, while the lowest was 0.5590 at the *TPOX* locus. For heterozygosity, the expected data ranged from 0.620 0 (*TPOX*) to 0.904 0 (*Penta E*), while the observed data ranged from 0.6214 (*TPOX*) to 0.920 6 (*Penta E*). In the Mongolia group, a total of 229 alleles were found, and the highest number of alleles (*N* = 19) was observed at *Penta E*. The highest PD value was 0.980 0 at *Penta E*, while the lowest was 0.7976 at the *TPOX* locus. The highest PIC value was 0.9117 at *Penta E*, while the lowest was 0.566 4 at the *TPOX* locus. For heterozygosity, the expected data ranged from 0.631 0 (*TPOX*) to 0.922 0 (*Penta E*) while the observed data ranged from 0.640 0 (*TPOX*) to 0.920 0 (*Penta E*).

Since the 23 STRs were independent from each other based on linkage disequilibrium testing, the cumulative power of exclusion in duos was 0.999 999 157 188, and the cumulative power of exclusion in trios was 0.999 999 999 859 in the Eastern Han group while the cumulative power of exclusion in duos (CPE_duo_) was 0.999 998 848 26 and cumulative power of exclusion in trios (CPE_trio_) was 0.999 999 999 79 in the Mongolia group. All the above data demonstrate that the 23 STRs are polymorphic in the two ethnic groups and can satisfy the forensic demands.

### Genetic analysis of 23 STRs

We also calculated the population pairwise genetic distance based on the allelic frequencies [10] between Eastern Chinese Han and other Chinese ethnic groups, including Mongolian (in this study), Northern Han [11], Sichuan Han [11], Guangdong Han [12], Hui [11], Tibetan [11], Uygur [11], She [13], Yi [14], Bai [15], Manchus [16], Tujia [17], Xibe [18], Ewenki [19], Zhuang [20], Dai [21], Dong [21], Kazak [22], Hani [23] and Miao [24]. Data are listed in Supplementary Table S3. The genetic distance ranged from −0.008 3 to 0.403 4; and the biggest Fixation index (*F*st) value was observed as 0.28745 at the *TPOX* locus between the Eastern Chinese Han and the Miao group, while the genetic distance was 0.4034 at this locus. Pairwise fixation index (*F*st) and the corresponding *P* values are listed as Supplementary Table S4. The *P* value of the test is the proportion of permutations leading to an Fst value larger or equal to the observed one. There were no significant differences between the Eastern Han and Mongolian/Tibetan/Yi/Ewenki groups at the compared loci (Supplementary Table S4); in other words, the allelic frequencies of the observed STRs are universal among these population groups. Within the 23 autosomal STRs, data from Eastern Chinese Han, Mongolian, Sichuan Han [11], Hui [11], Tibetan [11] and Uygur [11] were fully investigated, and the average genetic distances were all below 0.01, which indicate that these Chinese population groups exhibit good internal consistency at the included 23 STRs.

## Concluding remarks

The above results demonstrate that genotypes generated with the Early Access Huaxia™ Platinum PCR kit have been reproducible between laboratories and concordant with the existing STR-amplification systems. To obtain reliable genotype calling, at least 0.125 ng DNA or 1.2 mm^2^ samples should be prepared for PCR amplification. The allelic frequencies and forensic parameters support the efficacy and reliability of the system for forensic casework and/or database analysis. However, more optimization should be done for producing genotypes from challenging samples, including direct amplification from blood samples stored on FTA card or case-type samples. This report should serve as a demonstration of the capabilities and limitations of the Early Access Huaxia™ Platinum PCR kit.

## Compliance with ethical standards

All procedures performed in studies involving human participants were approved by the Ethics Committee of the Academy of Forensic Science and complied with the relevant national legislation and local guidelines.

## Supplementary Material

Supp_mat_TFSR.zip

## References

[1] Edwards A, Hammond HA, Jin L, et al. Genetic variation at five trimeric and tetrameric tandem repeat loci in four human population groups. Genomics. 1992;12:241–253. 1740333 10.1016/0888-7543(92)90371-x

[2] Butler JM Genetics and genomics of core short tandem repeat loci used in human identity testing. J Forensic Sci. 2006;51:253–265. 16566758 10.1111/j.1556-4029.2006.00046.x

[3] Moretti TR, Moreno LI, Smerick JB, et al. Population data on the expanded CODIS core STR loci for eleven populations of significance for forensic DNA analyses in the United States. Forensic Sci Int Genet. 2016;25:175–181. 27620707 10.1016/j.fsigen.2016.07.022

[4] Huang S, Zhu Y, Shen X, et al. Genetic variation analysis of 15 autosomal STR loci of AmpFlSTR Sinofiler PCR amplification kit in Henan (central China) Han population. Leg Med (Tokyo). 2010;12:160–161. 20303817 10.1016/j.legalmed.2010.02.004

[5] Huang D, Yang Q, Zhai X, et al. Allele frequencies and statistic parameters for penta D and penta E loci in Chinese Han population. J Forensic Sci. 2005;50:1515–1516. 16382863

[6] Zhang S, Bian Y, Tian H, et al. Development and validation of a new STR 25-plex typing system. Forensic Sci Int Genet. 2015;17:61–66. 25828368 10.1016/j.fsigen.2015.03.008

[7] Wang DY, Gopinath S, Lagacé RE, et al. Developmental validation of the GlobalFiler(®) Express PCR Amplification Kit: a 6-dye multiplex assay for the direct amplification of reference samples. Forensic Sci Int Genet. 2015;19:148–155. 26226223 10.1016/j.fsigen.2015.07.013

[8] Schlötterer C, Tautz D Slippage synthesis of simple sequence DNA. Nucleic Acids Res. 1992;20:211–215. 1741246 10.1093/nar/20.2.211 PMC310356

[9] Viguera E, Canceill D, Ehrlich SD In vitro replication slippage by DNA polymerases from thermophilic organisms. J Mol Biol. 2001;312:323–333. 11554789 10.1006/jmbi.2001.4943

[10] Rousset F Genetic differentiation and estimation of gene flow from F-statistics under isolation by distance. Genetics. 1997;145:1219–1228. 9093870 10.1093/genetics/145.4.1219 PMC1207888

[11] Wang Z, Zhou D, Jia Z, et al. Developmental validation of the Huaxia Platinum System and application in 3 main ethnic groups of China. Sci Rep. 2016;6:31075. 27498550 10.1038/srep31075 PMC4976323

[12] Chen L, Lu H, Qiu P, et al. Polymorphism analysis of 15 STR loci in a large sample of Guangdong (Southern China) Han population. Leg Med (Tokyo). 2015;17:489–492. 26593995 10.1016/j.legalmed.2015.10.001

[13] Yuan L, Ou Y, Liao Q, et al. Population genetics analysis of 38 STR loci in the She population from Fujian Province of China. Leg Med (Tokyo). 2014;16:314–318. 24996955 10.1016/j.legalmed.2014.05.008

[14] Zhu BF, Shen CM, Wu QJ, et al. Population data of 15 STR loci of Chinese Yi ethnic minority group. Leg Med (Tokyo). 2008;10:220–224. 18249578 10.1016/j.legalmed.2007.12.004

[15] Li Y, Hong Y, Li X, et al. Allele frequency of 19 autosomal STR loci in the Bai population from the southwestern region of mainland China. Electrophoresis. 2015;36:2498–2503. 26081075 10.1002/elps.201500129

[16] Liu J, Guo L, Qi R, et al. Allele frequencies of 19 autosomal STR loci in Manchu population of China with phylogenetic structure among worldwide populations. Gene. 2013;529:282–287. 23928110 10.1016/j.gene.2013.07.033

[17] Deng S, Cheng W [Genetic polymorphism of 15 STR loci in Chongqing Tujia population]. Chongqing Yi Ke Da Xue Xue Bao. 2009;34:1499–1501. Chinese.

[18] Guo L, Liu P, Qi R, et al. [Genetic polymorphism of fifteen STR loci of Xibe people in Liaoning]. Chongqing Yi Ke Da Xue Xue Bao. 2014;43:921–925. Chinese.

[19] Chen L, He Y, Li S Genetic analysis of 15 STR loci of Chinese Ewenki ethnic population. J Forensic Sci. 2006;51:1408–1409. 17199631 10.1111/j.1556-4029.2006.00279.x

[20] Liu X, He B, Yang Y, et al. [Genetic polymorphism of 17 STR loci in Nanning Zhuang population]. Guangxi Yi Xue. 2014;36:1360–1362. Chinese.

[21] Wang W, Sun Q, Zhang T, et al. [Genetic polymorphisms of 18 STR loci in Dai population of Yunnan and Dong population of Guangxi]. Shanxi Yi Ke Da Xue Xue Bao. 2015;46:991–995. Chinese.

[22] Liu S, Jia F, Liu F [Genetic polymorphism of 23 STR loci of Kazak population in Hotan area]. Xing Shi Ji Shu. 2015;40:422–423. Chinese.

[23] Li ZJ, Wu H, Wu HJ, et al. [Genetic polymorphism of 17 STRs in Hani and Han population of Suzhou]. Fa Yi Xue Za Zhi. 2015;31:152–155. Chinese.

[24] Zhang W, Huang L, Xiang C, et al. [Genetic polymorphisms of 17 STR loci in Miao population of Yunnan]. Zhong Guo Fa Yi Xue Za Zhi. 2016;31:181–182. Chinese.

