# Supplementary material for: Forensic investigation of 23 autosomal STRs and application in Han and Mongolia ethnic groups
Source: Forensic Sci Res. 2018 Apr 2;3(2):138–44. doi: 10.1080/20961790.2018.1428782 (PMC6197138; doi:10.1080/20961790.2018.1428782)
Supplement: Supp_mat_TFSR.zip [file TFSR_A_1428782_SM5447.zip › Supp_mat_TFSR/Supplementary.doc]

**Supplementary Figures S1-S2 and Supplementary Tables S1-S4**

**Forensic investigation of 23 autosomal STRs and application in HAN and MONGOLIA ethnic groups**

Xiang Sheng^1,2+^, Yali wang^1,3+^, Jiashuo Zhang^1,2^, Liqin Chen^3^, Yuan Lin^1^, Zhenmin Zhao^1^, Chengtao Li^1^*, Suhua Zhang^1^*

^1^ Shanghai Key Laboratory of Forensic Medicine, Shanghai Forensic Service Platform, Academy of Forensic Sciences, Ministry of Justice, P.R. China, Shanghai 200063, P.R. China

^2^ Department of Forensic Medicine, Medical College of Soochow University, Suzhou 215123, P.R. China

^3^ Department of Forensic Medicine, Inner Mongolia Medical University, Hohhot, 010030, P.R. China

*Corresponding author.

Corresponding authors: Chengtao Li and Suhua Zhang (ORCID: 0000-0002-3984-9728)

E-mail: [zsh-daisy@163.com](mailto:lichengtaohla@163.com)


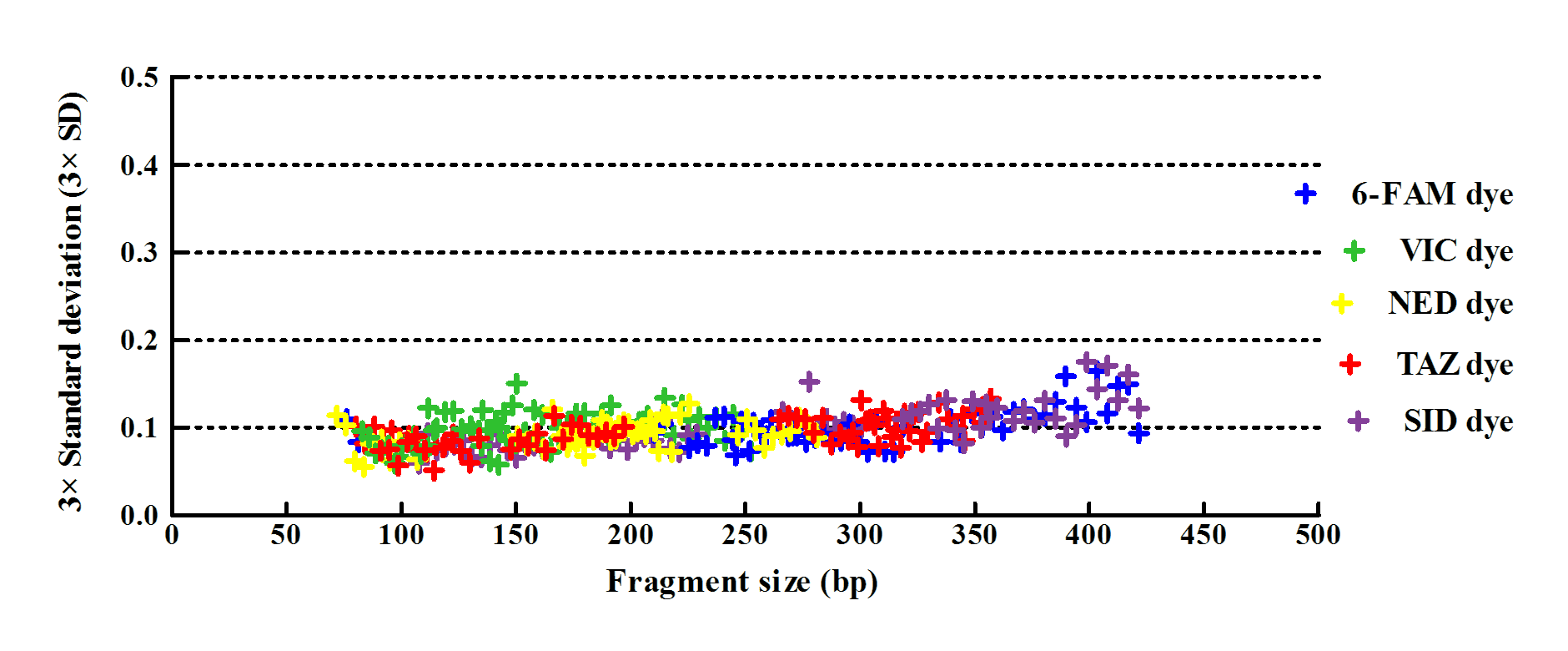


Supplementary Fig S1 Sizing precision testing across 24 injections of the allelic ladder of Early Access Huaxia^TM^ Platinum PCR kit performed on 3500xl Genetic Analyzer.


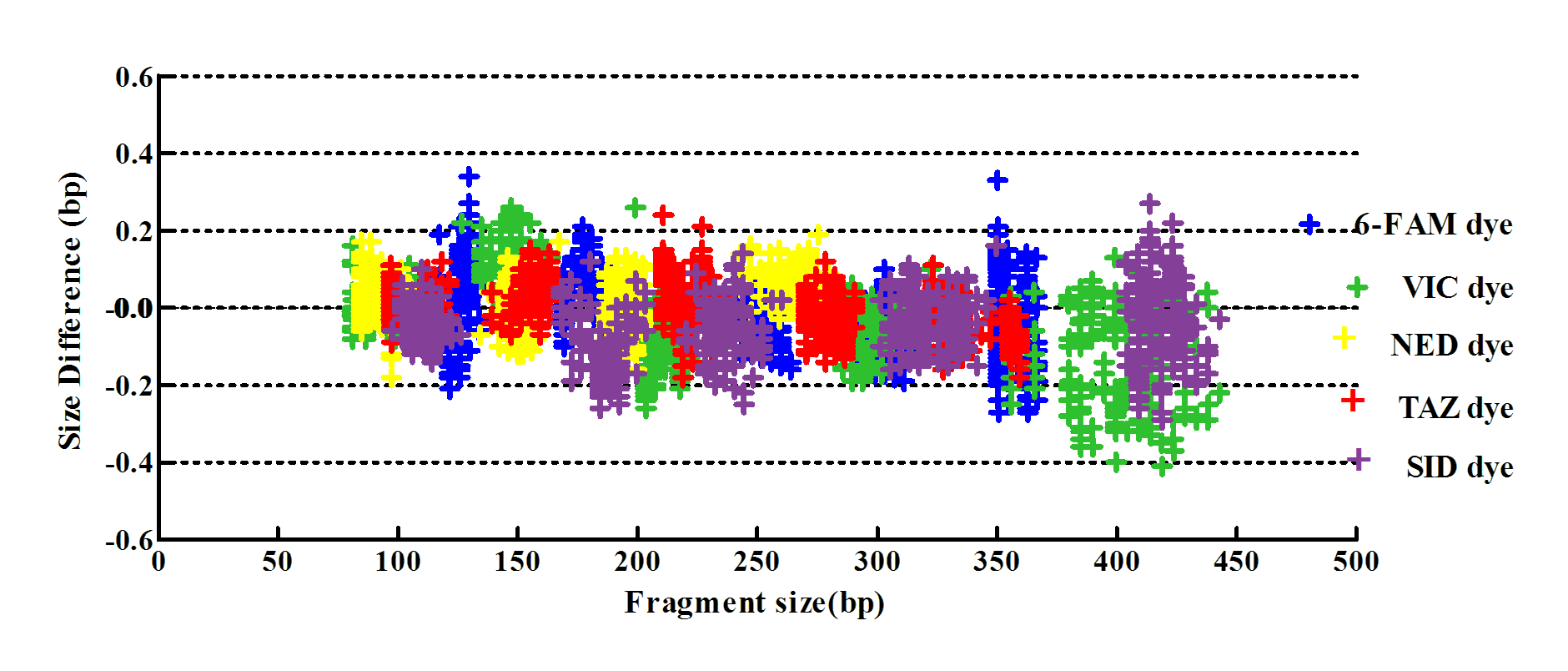


Supplementary Fig S2 Size differences between each sample allele (n=4053) and corresponding allelic ladder allele.

Supplementary Tables S1 Sample information for validation studies including sensitivity, reliability and repeatability, sizing precision and accuracy, stutter analysis, case sample study, mixture study and population investigation

Supplementary Table S2 Forensic parameters of the included 23 autosomal STRs in Eastern Chinese HAN and MONGOLIA ethnic groups

Supplementary Table S3 Genetic distances of 23 autosomal STRs between Eastern Chinese HAN and other Chinese ethnic populations

Supplementary Table S4 Fst and p values of 23 autosomal STRs between Eastern Chinese HAN and other Chinese ethnic populations with available reference data
